# Scaling Law for Irreversible Entropy Production in Critical Systems

**DOI:** 10.1038/srep27603

**Published:** 2016-06-09

**Authors:** Danh-Tai Hoang, B. Prasanna Venkatesh, Seungju Han, Junghyo Jo, Gentaro Watanabe, Mahn-Soo Choi

**Affiliations:** 1 Asia Pacific Center for Theoretical Physics (APCTP), Pohang, Gyeongbuk 37673, Korea; 2 National Institute of Diabetes and Digestive and Kidney Diseases, National Institutes of Health, Bethesda, Maryland 20892, USA; 3 Department of Natural Sciences, Quang Binh University, Dong Hoi, Quang Binh 510000, Vietnam; 4 Institute for Quantum Optics and Quantum Information of the Austrian Academy of Sciences, Technikerstraße 21a, Innsbruck 6020, Austria; 5 Institute for Theoretical Physics, University of Innsbruck, A-6020 Innsbruck, Austria; 6 Department of Physics, Korea University, Seoul 02841, Korea; 7 Department of Physics, Pohang University of Science and Technology (POSTECH), Pohang, Gyeongbuk 37673, Korea; 8 Center for Theoretical Physics of Complex Systems, Institute for Basic Science (IBS), Daejeon 34051, Korea; 9 University of Science and Technology (UST), 217 Gajeong-ro, Yuseong-gu, Daejeon 34113, Korea; 10 Department of Physics, Zhejiang University, Hangzhou, Zhejiang 310027, China

## Abstract

We examine the Jarzynski equality for a quenching process across the critical point of second-order phase transitions, where absolute irreversibility and the effect of finite-sampling of the initial equilibrium distribution arise in a single setup with equal significance. We consider the Ising model as a prototypical example for spontaneous symmetry breaking and take into account the finite sampling issue by introducing a tolerance parameter. The initially ordered spins become disordered by quenching the ferromagnetic coupling constant. For a sudden quench, the deviation from the Jarzynski equality evaluated from the ideal ensemble average could, in principle, depend on the reduced coupling constant *ε*_0_ of the initial state and the system size *L*. We find that, instead of depending on *ε*_0_ and *L* separately, this deviation exhibits a scaling behavior through a universal combination of *ε*_0_ and *L* for a given tolerance parameter, inherited from the critical scaling laws of second-order phase transitions. A similar scaling law can be obtained for the finite-speed quench as well within the Kibble-Zurek mechanism.

Fluctuation theorems (FTs) provide universal and exact relations for nonequilibrium processes irrespective of how far a system is driven away from equilibrium. The discovery of FTs is a major development in nonequilibrium statistical mechanics, pioneered by Bochkov and Kuzovlev^1^,^2^ for a special case and thriving with the celebrated equalities of Jarzynski^3^ and Crooks^4^ which hold for general forcing protocols (see, e.g.,^5^,^6^,^7^,^8^ and references therein for recent reviews).

Since the discoveries of the Jarzynski equality (JE) and the Crooks relation, a large effort has been made to find applications of these universal relations. As a representative example, FTs provide a unique way to evaluate the free energy difference Δ*F* between equilibrium states through nonequilibrium processes^3^, which could be useful for systems such as complex molecules^9^,^10^ that take a very long time to reach an equilibrium state. FTs have also been exploited to study the nonequilibrium dynamics^11^,^12^,^13^,^14^, to show the emergence of thermodynamics out of microscopic reversibility^15^, and to investigate the universal behaviors of the work-distribution tails^16^ in quantum critical systems. Further, FTs by themselves serve as useful formulae which simplify theoretical derivations and facilitate important developments such as information thermodynamics^17^.

Although the FTs hold universally, they require sufficient sampling from the initial ensemble, causing a *convergence problem* in many situations^18^,^19^,^20^,^21^,^22^. For example, consider the JE, 〈*e*^−*σ*^〉 = 1, where *σ* = *β*(*W* − Δ*F*) is the irreversible entropy production, *W* the work performed to the system, and *β* the inverse temperature. The realizations of a thermodynamic process which yield the dominant contribution to the ensemble average of *e*^−*σ*^ can be very different from typical realizations under the same condition. Then, sufficient sampling of the dominant realizations becomes intractable with increasing system size, and in reality the JE is hard to verify to high accuracy with a finite number of samples.

Moreover, even in the ideal case with sufficient sampling, there are a class of processes such as the free expansion of a gas, to which the JE does not apply due to a fundamental reason that has been referred to as *absolute irreversibility*^23^,^24^,^25^,^26^,^27^,^28^. A process is called absolutely irreversible if there exists a path in phase space whose probability to occur in the forward direction is zero while that in the reverse direction is nonzero, or vice versa. A typical situation occurs when the accessible phase spaces for the system at the beginning and end of a protocol are not identical. This is indeed the case for the free expansion of initially confined particles whose accessible phase space is increased by removing the partitioning barrier.

In this work, we explore the fact that in systems driven through second-order phase transitions, both the absolute irreversibility and the convergence issue can take place in a single setup with equal significance. Using the scaling theory of phase transitions and numerical simulations, the deviation from the *ideal ensemble average* JE, i.e., the ensemble average of *e*^−*σ*^ using infinite number of samples, is examined as a function of the system size and the reduced coupling constant. It exhibits a universal scaling behavior inherited from the critical scaling of the correlation length and the relaxation time in second-order phase transitions. This finding may provide a unique application of the FTs to study the dynamical properties of phase transitions. We note that the absolute irreversibility due to the spontaneous symmetry breaking is analogous to the ergodicity loss (within a finite observation time) in a *finite* system with metastable states. The latter has been explored theoretically by assuming partially equilibrated states^29^,^30^, and demonstrated in an experiment with a Brownian particle subject to a bistable potential of two optical traps^31^ or similar experiments^32^,^33^ in the context of Landauer’s principle. In our work, we explore how the ergodicity breaking intensifies with the system size and reveal that the effects of spontaneous symmetry breaking are reflected in the scaling behavior of the deviation from the ideal ensemble average JE.

While the detailed arguments and analyses are discussed below, a summary of our discussion is as follows: On the one hand, a natural partitioning of the phase space emerges as a consequence of the ergodicity breaking in the ordered phase^34^ in contrast to the partitioning externally imposed in the example of free expansion. The resultant absolute irreversibility is illustrated in Fig. 1 for the Ising model, which as the simplest model showing spontaneous symmetry breaking (SSB) will be used to anchor the rest of our discussions. It is expected that the SSB of 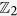 symmetry corresponds to halving the accessible phase space, resulting in 〈*e*^−*σ*^〉 = 1/2 when the system is quenched from the equilibrium ordered to the disordered phase. On the other hand, in such a process the configurations with vanishing (spatial) mean order parameter give major contributions to 〈*e*^−*σ*^〉, while such configurations are extremely rare in the initial equilibrium in the ordered phase. Here, such an insufficient sampling is accounted for by introducing a tolerance parameter to neglect some unlikely configurations and in general this leads to lower value of 〈*e*^−*σ*^〉 than for the ideal case. We stress that an observation over a finite time in realistic experiments and numerical simulations inevitably leads to insufficient sampling. Our main result is that for a given tolerance the deviation from the ideal ensemble average JE, neither zero nor exactly one half, is still determined in terms of a universal combination of the reduced coupling constant and the system size.

## Model

We take the Ising model as the simplest example showing SSB. It consists of *N* = *L*^*d*^ spins 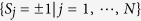 on a *d*-dimensional lattice of lateral size *L* whose interaction is governed by the Hamiltonian


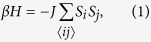


where 〈*ij*〉 denote pairs of nearest-neighbor sites and *J* the coupling strength. We denote a spin configuration by 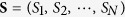, its magnetization per spin by


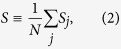


and the set of all spin configurations by 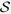. The configuration space 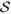 consists of 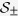 and 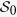, 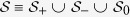, where


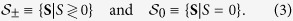



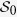
 is naturally empty for odd *N*. For even *N*, 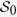 is negligible (probability measure is zero) in the thermodynamic limit and hereafter ignored.

We consider, for simplicity and to conform to the standard protocols of the JE, quenching processes where the coupling constant *J*(*t*) varies while temperature is kept constant. As usual, we define the *reduced coupling constant* by


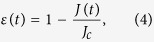


where *J*_*c*_ is the critical point. As the time *t* changes from *t*_*i*_ to *t*_*f*_, *ε*(*t*) changes from *ε*_*i*_ ≡ *ε*(*t*_*i*_) to *ε*_*f*_ ≡ *ε*(*t*_*f*_) and *H*(*t*) from *H*_*i*_ ≡ *H*(*t*_*i*_) to *H*_*f*_ ≡ *H*(*t*_*f*_). To discuss absolute irreversibility, we will be mostly interested in quenching from the ordered (*ε*_*i*_ < 0) to disordered (*ε*_*f*_ > 0) phase. For simplicity, we consider symmetric quenching: *ε*_*f*_ = −*ε*_*i*_ = *ε*_0_ > 0.

Although the quenching process drives the system out of equilibrium, many physical effects are still described in terms of the initial and final *equilibrium* distribution functions


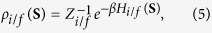


where *Z*_*i*/*f*_ are the respective partition functions. Since we start from the ordered phase at initial time, the allowed spin configurations are restricted either to 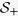 or 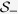 due to SSB. For keeping the discussion specific we take the spin configurations to be in 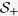 giving the initial partition function 
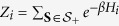
 while the final partition function is given by 
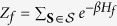
 as usual. We will see that the restriction of the initial spin configurations has vital consequences. For large yet finite systems in the ordered phase, the free energy barrier is still high enough to make the transitions between 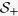 and 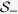 very rare, practically never happen at time scales sufficiently shorter than the ergodicity-breaking time (EBT)^34^. As long as the equilibrium state is concerned, *ρ*_*i*_ can be safely confined within either 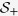 or 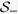. Note that typical systems exhibiting phase transitions have the EBT much larger than the observation time. We also note that the equilibrium probabilities


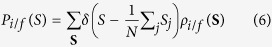


of magnetization per spin *S* are particularly useful.

## Absolute Irreversibility in the Ising Model

We first illustrate that for the Ising model 〈*e*^−*σ*^〉 = 1/2, regardless of how the parameter is tuned in time. We remark that the argument here is exact.

Following the previous work^28^, we characterize the break-down of the Jarzynski equality as


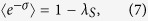


where *λ*_*S*_ is the probability of the absolutely irreversible paths, i.e., the total probability of backward paths for which the corresponding forward paths are absent.

Let 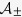 be the subset of 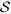 that cannot be reached by any forward path Γ_fwd_ from 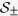. Obviously, 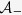 is the symmetric counterpart of 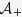 as 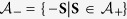. Let 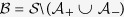 denote the set of spin configurations that can be reached by some Γ_fwd_ starting from either 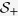 or 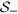 or both. Figure 2 summarizes the relation among these sets.

Now we note that, by definition, any backward path Γ_bwd_ starting from 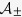 will end up necessarily in 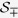 while Γ_bwd_ from 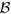 may arrive at either 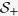 or 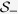. This can be expressed as


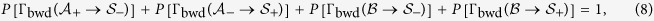


where 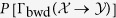 the probability for a backward path Γ_bwd_ starting from the set 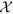 of spin configurations to reach the set 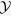. From the symmetry we see that


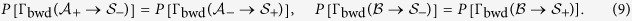


It implies that the probability of backward paths whose corresponding forward paths (starting from 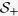) do not exist is given by


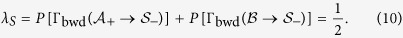


We thus conclude that 〈*e*^−*σ*^〉 = 1/2 for arbitrary quench protocols varying the parameter *ε*(*t*).

## Tolerance Parameter

Here we introduce a tolerance parameter to account for the insufficient sampling, which is inevitable in experiments and numerical simulations. In addition, a properly defined tolerance parameter enables to study the dynamical properties of phase transitions as we shall see in the remaining part of this paper.

In the so-called “sudden” (infinitely fast) quenching (more general cases are discussed below), the system does not have enough time to change its distribution over spin configurations, and hence the initial equilibrium distribution is preserved throughout the whole process. The work distribution is thus completely determined by *ρ*_*i*_, leading to


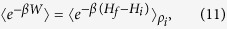


where the average 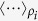 is over the initial distribution *ρ*_*i*_(**S**). Recall that the initial spin configurations are restricted to 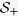 due to the SSB. The exponential average of the entropy production *σ* = *β*(*W* − Δ*F*) follows easily from 〈*e*^−*βW*^〉 by multiplying by the exponential of the free energy change given by *β*Δ*F* = −log(*Z*_*f*_/*Z*_*i*_). In realistic experiments and numerical simulations, spin configurations with exponentially small probability do not take actual effects. Therefore it is natural to ignore such spin configurations up to certain tolerance *δ*. (Also practically, configurations with very low probabilities that cannot be sampled sufficiently, have to be discarded in the actual analysis of experiments and numerical simulations.) Specifically, for a given probability distribution *ρ* and tolerance *δ*, we *implicitly* define the set of kept spin configurations 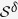 and the cutoff probability *ρ*_cut_ by the following two conditions (see also Fig. 3):


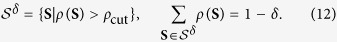


We introduce the short-hand notations 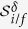 for the initial/final configurations *ρ*_*i*/*f*_(**S**). The corresponding partition functions are given by 
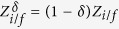
, leading to


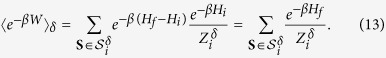


The free energy change is not affected by tolerance, 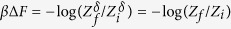. We thus obtain


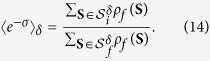


**Figure 1:**
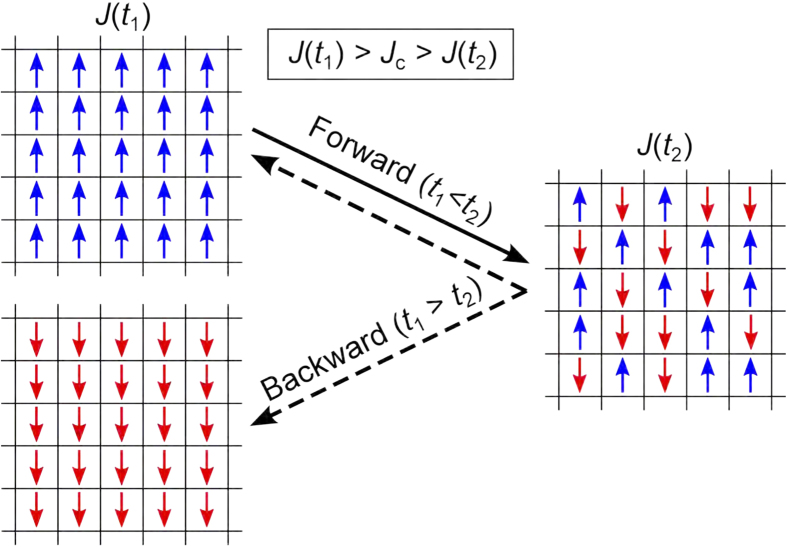
Schematic representation of the absolute irreversibility in the quench dynamics of Ising model. In the forward process, the system is initially at equilibrium with positive spontaneous magnetization, whereas in the backward process the initial equilibrium state has no magnetization. When the coupling *J* increases across the critical point, the system can have either positive or negative magnetization. The latter case has no corresponding forward path, which results in the absolute irreversibility.

**Figure 2:**
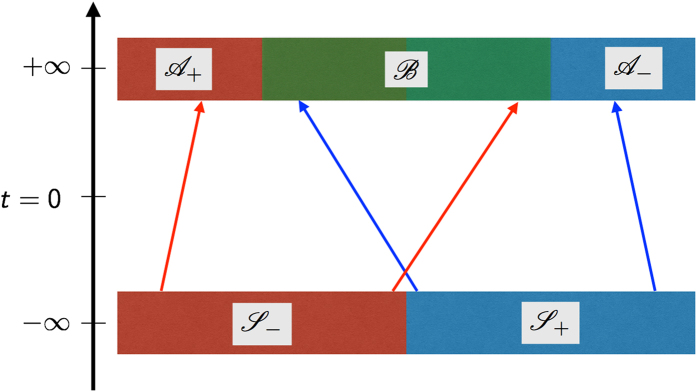
Possible forward paths and images of them.

**Figure 3:**
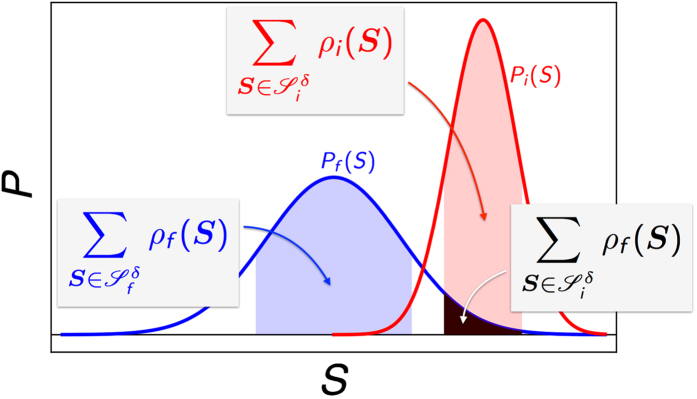
Schematic representation of the sets 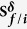 of allowed spin configurations and their relations to 〈*e*^−*σ*^〉_*δ*_ . For a given tolerance *δ*, 〈*e*^−*σ*^〉_*δ*_ is given by the ratio of the areas in black and blue shade.

Equation (14) is one of our main results and manifests several features to be stressed^29^,^30^: (i) As illustrated schematically in Fig. 3, 〈*e*^−*σ*^〉_*δ*_ depends crucially on the overlap of 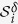 and 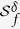. For finite *δ*, well separated initial and final distributions lead to vanishing 〈*e*^−*σ*^〉_*δ*_. For *δ* = 0, on the other hand, 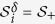 and 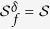, and hence 〈*e*^−*σ*^〉 = 1/2 validating the heuristic analysis presented in Fig. 1. (ii) Equation (14) also demonstrates how the convergence issue arises in quenching process of phase transitions. Namely, the dominant contributions to the ensemble average of *e*^−*σ*^ comes from the spin configurations with larger *ρ*_*f*_(**S**) whereas the initial equilibrium is governed by those with larger *ρ*_*i*_(**S**). (iii) Equation (14) describes highly *nonequilibrium* processes merely in terms of *equilibrium* distributions, a remarkably simple way to study 〈*e*^−*σ*^〉_*δ*_.

The tolerance scheme (12) in terms of the *microscopic* spin configurations **S** is still difficult to implement in practice. For example, the tolerance parameter *δ* corresponding to the actual finite sampling is unknown or very difficult to estimate in most cases. For this reason, we introduce another *operational* tolerance scheme in terms of the *macroscopic* order parameter *S*: Given *P*_*μ*_(*S*) (*μ* = *i*, *f*), we *implicitly* define the interval of relevant magnetization 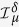 and the cutoff *P*_cut_ by


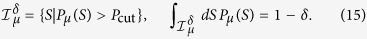


Note that the relation (14) does not depend on a particular tolerance scheme, and for the scheme (15) it reads as


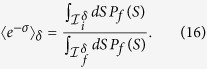


Below we will mainly use the tolerance scheme (15); the relation between the two tolerance scheme is discussed in the Supplementary Information.

## Universal Scaling Behavior

We now examine 〈*e*^−*σ*^〉_*δ*_ in Eq. (16) more closely with numerical simulations and analytical arguments, focusing on its scaling behavior inherited from the spontaneous symmetry breaking. According to Eq. (16), in the case of sudden quench it is enough to calculate the equilibrium distributions *P*_*i*_(*S*) and *P*_*f*_(*S*); no need to simulate the quenching dynamics.

To calculate the probability distribution of magnetization per spin, *P*(*S*), we perform a Monte Carlo simulation. Briefly, we (i) randomly generate initial spin configurations; (ii) choose one spin for flip at random and calculate the energies *βH* and *βH*′ in Eq. (1) before and after the flip; (iii) accept the flip with a probability, min[1, exp(*βH* − *βH*′)], following the Metropolis algorithm^35^; (iv) repeat these procedures in several million Monte Carlo steps per spin to achieve sufficient equilibration; (v) and after the equilibration, finally perform another 10 to 20 millions of Monte Carlo steps per spin to obtain the distribution of the order parameter *S* in Eq. (2). Here we consider two-dimensional (2D) square lattices of size *L*^2^ = 50^2^, 100^2^, and 200^2^; and three-dimensional (3D) cubic lattices of size *L*^3^ = 20^3^, 40^3^, and 50^3^.

### Scaling Law

**Figure 4:**
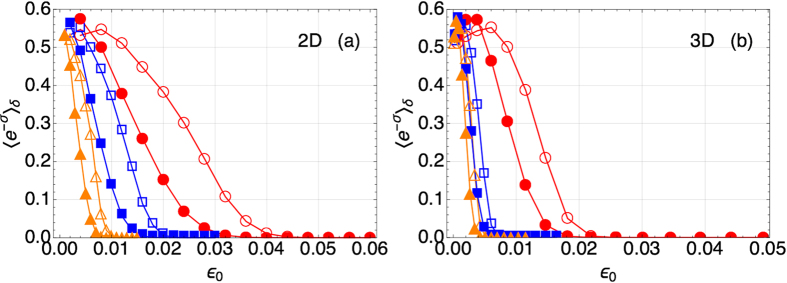
〈*e*^−*σ*^〉_*δ*_ as a function of *ε*_0_ from Monte Carlo simulations of the Ising model, for *δ* = 0.1 (empty symbols) and *δ* = 0.3 (filled symbols). (**a**) On a 2D square lattice with *L* = 50 (circles), *L* = 100 (squares), and *L* = 200 (triangles). (**b**) On a 3D cubic lattice with *L* = 20 (circles), *L* = 40 (squares), and *L* = 50 (triangles).

**Figure 5:**
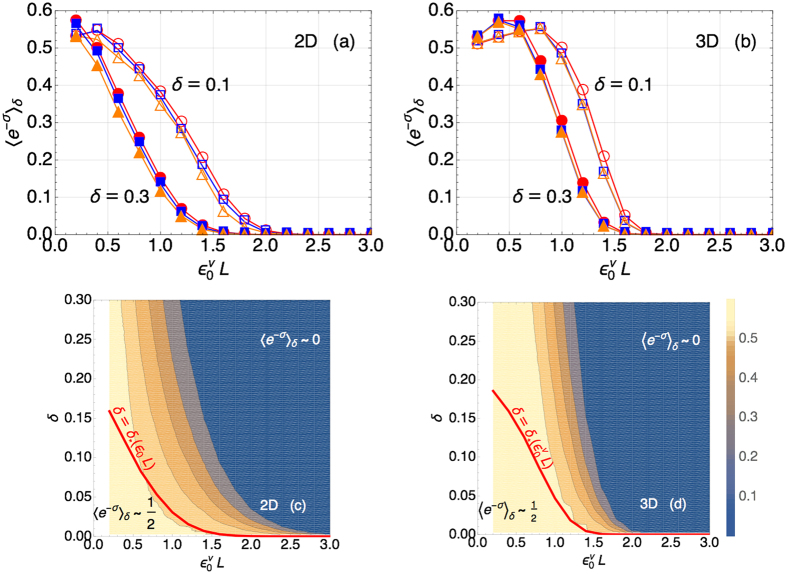
Scaling law in 〈*e*^−*σ*^〉_*δ*_. (**a**,**b**) The same as Fig. 4 but as a function of the universal scaling combination 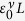. (**c**,**d**) The contour plot of 〈*e*^−*σ*^〉_*δ*_ as a function of 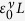 and *δ*. The thick red line represents the crossover boundary, 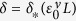.

We calculate the distributions *P*_*i*/*f*_(*S*) upon quenching the coupling constant (*ε*_*i*_ = −*ε*_0_ → *ε*_*f*_ = *ε*_0_) across the critical point (*ε* = 0). Based on Eq. (16), we obtain 〈*e*^−*σ*^〉_*δ*_ as a function of *ε*_0_ and *L* for some representative values of *δ* = {0.1, 0.3}. Figure 4 displays the results of such a calculation and we can immediately see that for both 2 and 3 dimensional lattices 〈*e*^−*σ*^〉_*δ*_ decreases as *ε*_0_ is increased (at fixed *L*) or as *L* is increased (at fixed *ε*_0_). Most remarkably, for a given tolerance *δ*, we find that 〈*e*^−*σ*^〉_*δ*_ does not depend on *ε*_0_ and *L* separately, but unexpectedly it is a universal function of 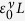 with an exponent *ν* as shown in Fig. 5(a,b). Moreover we find that the exponent *ν* is nothing but the scaling exponent of the correlation length 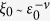 at *ε* = *ε*_0_ giving 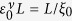 with *ν* = 1 in 2D and *ν* = 0.6301 in 3D^36^. The discovery of this universal scaling behavior is the central result of the paper. In what follows we justify this universal behaviour with analytical reasoning based on the scaling theory of second order phase transitions.

According to Eq. (16), the overlap between the distribution functions *P*_*μ*_(*S*) (*μ* = *i*, *f* ) plays a crucial role in 〈*e*^−*σ*^〉_*δ*_. Let us investigate this overlap based on the scaling analysis here (and the large deviation theory below). For sufficiently large systems, the distributions are rather sharp and it suffices to characterize them by the peaks 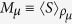 (recall that *M*_*f*_ = 0) and their widths 
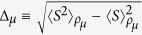
. According to the fluctuation-dissipation theorem^34^, Δ_*μ*_ is related to the equilibrium susceptibility *χ*_*μ*_ by 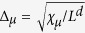. Note that beyond the above assumption to characterize the distributions using their peaks and widths, we do not assume any specific functional form of the distributions. Notably, the distribution can be highly non-Gaussian for small systems^37^ and/or for systems with a *continuous* symmetry broken spontaneously^38^.

One can understand the arguments more rigorously along the lines of the large deviation theory^16^,^37^,^39^,^40^,^41^,^42^,^43^,^44^: For sufficiently large systems (*N* = *L*^*d*^ → ∞), the probability distributions behave as


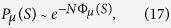


where


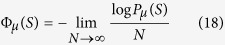


is the so-called rate function. Thermodynamically, Φ_*μ*_(*S*) can be expressed in terms of the Helmholtz free energy density *F*_*μ*_(*B*) and the Gibbs free energy density *G*_*μ*_(*S*) by^37^,^34^


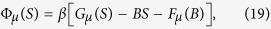


where *B* is the external magnetic field. *G*_*μ*_(*S*) is the Legendre-Fenchel transformation of *F*_*μ*_(*B*), *G*_*μ*_(*S*) = sup_*B*_[*F*_*μ*_(*B*) + *BS*], which reduces to the usual Legendre transformation above the critical temperature. The saddle-point approximation gives the leading behaviors of the rate functions^39^,^41^


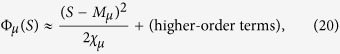


which are consistent with the above arguments. This approximation becomes accurate in the thermodynamic limit and explored further below [Eqs (28, 29, 30, 31, 32)] to get an analytical universal expression of 〈*e*^−*σ*^〉_*δ*_ in the limit. The universal tails of the work distribution near a *quantum* critical point has been revealed based on the large deviation theory^16^. Hence, investigating the overlap between *P*_*i*_(*S*) and *P*_*f*_(*S*) (and 〈*e*^−*σ*^〉_*δ*_ in turn) more closely by means of the large deviation theory will be an interesting issue on its own, and we leave it open for future work. Instead, here we characterize the overlap by introducing the *relative separation* of the peaks of *P*_*i*_(*S*) and *P*_*f*_(*S*), 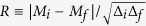.

The magnetization and susceptibility satisfy the standard scaling behaviors:


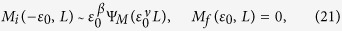



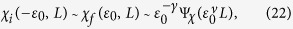


where *β* and *γ* are the critical exponents, and Ψ_*M*/*χ*_(*z*) are the universal scaling functions. The scaling functions asymptotically approach Ψ_*M*/*χ*_(*z*) = 1 for *z* → ∞ while Ψ_*M*_(*z*) ~ *z*^−*β*/*ν*^ and Ψ_*χ*_(*z*) ~ *z*^*γ*/*ν*^ for *z* → 0. Here, for simplicity we have ignored the irrelevant difference in Δ_*i*_ = Δ_*f*_ = Δ (*χ*_*i*_ = *χ*_*f*_ = *χ*) above and below the critical point. Putting Eqs (21) and (22) together with the Rushbrooke scaling law^34^, *α* + 2*β* + *γ* = 2, one has the relative separation


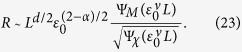


Using the Josephson hyperscaling law^34^, *dν* = 2 − *α*, it is further reduced to


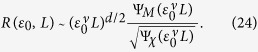


It is remarkable that the relative separation 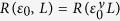 does not depend on *ε*_0_ and *L* separately but is a universal function of only the combination 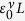. This implies that 〈*e*^−*σ*^〉_*δ*_ is also a universal function of 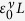 alone, which is indeed confirmed by the numerical results shown in Fig. 5(a,b). Note that the hyperscaling law breaks down either in dimensions higher than the upper critical dimension d_*_ = 4 or in the mean-field approximation. In such cases, where *α* = 0 and *ν* = 1/2, 〈*e*^−*σ*^〉_*δ*_ is not necessarily a universal function of 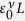 in general.

For sufficiently large systems 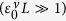, one expects sharp distribution functions. Indeed, in this limit it follows that


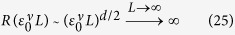


and *P*_*i*/*f*_(*S*) are well separated. On the other hand, when the system is small 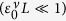 and finite-size effect sets in, the larger fluctuations lead to broader distribution functions giving


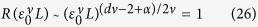


according to the hyperscaling law. It means that *P*_*i*/*f*_(*S*) have significant overlap with each other for a finite-size system. With the universal scaling behaviors of relative separation *R* at hand, let us now investigate 〈*e*^−*σ*^〉_*δ*_. For a given tolerance *δ*, the two asymptotic behaviors in Eqs (25) and (33) imply little and significant overlap between *P*_*i*/*f*_(*S*), respectively, and hence that 〈*e*^−*σ*^〉_*δ*_ ~ 0 in the limit of 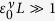, while 〈*e*^−*σ*^〉_*δ*_ ~ 1/2 in the opposite limit of 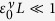 using the intuition provided by Eq. (16) and Fig. 3.

### Effect of Tolerance

Ideal sampling (*δ* = 0) always gives 〈*e*^−*σ*^〉_*δ*_ = 1/2. However, for 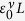 fixed, large tolerance (*δ* ~ 1) naturally leads to 〈*e*^−*σ*^〉_*δ*_ ~ 0, because it decreases the overlap between the initial and final distributions *P*_*i*/*f*_ (*S*) in Fig. 3. In the parameter space of 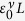 and *δ*, 〈*e*^−*σ*^〉_*δ*_ ~ 1/2 in the limit 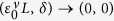, while 〈*e*^−*σ*^〉_*δ*_ ~ 0 in the opposite limit of 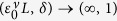 as depicted in Fig. 5(c,d).

Evidently, a crossover of 〈*e*^−*σ*^〉 occurs as a result of combined effects of finite size and tolerance. One can locate the crossover boundary 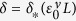 by identifying δ_*_ for given 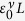 as the maximum tolerance allowing for significant overlap between 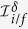. More specifically, δ_*_ is such that the lower end of the interval 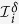 (i.e., min 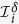; recall that *M*_*i*_ > 0) equals to the center (i.e., *M*_*f*_ = 0) of 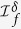:


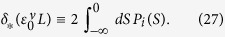


The crossover boundaries are illustrated by the thick red lines in Fig. 5(c,d). Here we note that the universality of 〈*e*^−*σ*^〉_*δ*_ as a function of 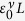 is valid up to the critical scaling of phase transitions. In the critical region, any physical quantity has both regular and singular parts, and only the singular part exhibits the scaling behavior. Therefore, 〈*e*^−*σ*^〉_*δ*_ can also in general have a regular part, which does not obey the universal dependence on 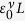. Indeed, for fixed *δ*, Fig. 5(a,b) show three slightly different curves for different values of *L*. In Fig. 5(c,d) the contours plot 〈*e*^−*σ*^〉_*δ*_ averaged over different values of *L*.

### Thermodynamic Limit

Figure 5 shows that 〈*e*^−*σ*^〉_*δ*_ is suppressed exponentially for sufficiently large systems 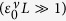 while it recovers 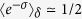 for small systems 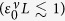 for a given tolerance *δ*. [In the presence of small tolerance, 〈*e*^−*σ*^〉_*δ*_ can slightly exceed 1/2, due to the renormalization of the distribution functions *P*_*i*/*f*_(*S*) truncated in accordance with the tolerance parameter.] In the thermodynamic limit 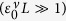, 〈*e*^−*σ*^〉_*δ*_ can be investigated more closely because the distributions *P*_*i*/*f*_(*S*) are sharp and take the Gaussian form [see Eq. (20)]


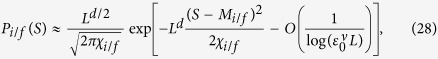


where *M*_*f*_ = 0. The crossover tolerance in Eq. (27) becomes 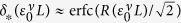, where 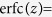

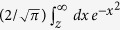
 is the complementary error function. Using the asymptotic behavior 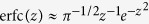 at *z* → ∞, it can be further simplified as


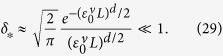


Therefore, in practice 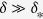 always and 〈*e*^−*σ*^〉_*δ*_ tends to vanish in the thermodynamic limit. Using Eq. (16), one can examine the tendency more closely:


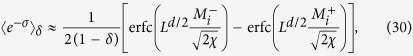


where 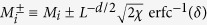 defines the interval 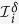 by means of the relation


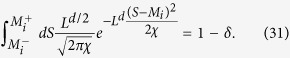


Since 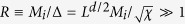 for 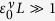 in Eq. (25), one can obtain


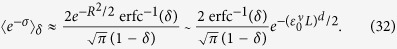


Thus, the exponential of entropy production at finite tolerance exponentially vanishes in the thermodynamic limit.

## Finite-Speed Quenching

So far we have examined the nonequilibrium dynamics of the entropy production for the sudden quenching of the reduced coupling. Here we discuss the more general case of a finite-speed quench. As we already illustrated at the beginning, for zero tolerance one still has 〈*e*^−*σ*^〉 = 1/2 exactly. Now we examine the case with a finite tolerance. We will employ the Kibble-Zurek mechanism (KZM) and demonstrate that the nonequilibrium dynamics of 〈*e*^−*σ*^〉_*δ*_ reflects the equilibrium scaling properties of the system even in this case. Recall that due to the convergence issue in large systems, the numerical simulation for the finite-speed quench is very demanding computationally. The analysis based on the KZM may provide an outlook.

We consider a linear quenching process where the coupling constant varies in time as *ε*(*t*) = *ct* for *t* ∈ (−∞, ∞) with the finite quenching speed *c*. We assume a finite tolerance *δ*, up to which relatively improbable spin configurations are ignored.

In general, the nonequilibrium dynamics caused by such a quench can be very complicated. Here we adopt the spirit of the KZM and make the adiabatic-impulse approximation. The KZM was originally put forward to study the cosmological phase transition of the early Universe^45^,^46^ and later extended to study classical phase transitions in condensed matter systems^47^,^48^. Recently, it was also found to apply to the Landau-Zener transitions in two-level quantum systems^49^,^50^ and the dynamics of second-order quantum phase transitions^51^,^52^. As a theory of the formation of topological defects in second-order phase transitions, the KZM establishes accurate connections between the equilibrium critical scalings and the nonequilibrium dynamics of symmetry breaking.

The key idea of the KZM is to classify the dynamics into two distinct regions, the adiabatic and impulse regimes. Recall that, in a second-order phase transition, the relaxation time *τ* provides the single time scale analogous to the correlation length *ξ* which is the only length scale of the system. The former is naturally related to the latter by


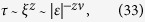


where *z* is the dynamic critical exponent, and diverges at the critical point (*ε* = 0). In the early stage of the linear quenching process (*t* → −∞), the system is far from the critical point, and its relaxation time *τ*(*t*) ~ |*ε*(*t*)|^−*zν*^ → 0 is short enough so that it can adjust itself to the change in the reduced coupling actuated by the driving. In this sense, the dynamics is adiabatic. On the contrary, as the system approaches the critical point (*ε*(*t*) → 0), the relaxation time *τ* diverges and the relaxation of the system is too slow to follow the external change. The system essentially remains the same and the external driving can be regarded as impulsive. In the far future (*t* → ∞), getting away from the critical point, the relaxation time decreases back and the dynamics becomes adiabatic again. The crossover between the two dynamical regimes occurs at *t* = ±*t*_*c*_ when the relaxation time becomes comparable with the time scale, 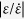, for *ε*(*t*) to develop, namely, 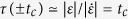. It follows from (33) that *t*_*c*_ ~ *c*^−*zν*/(1+*zν*)^ and *ε*(*t*_*c*_) ~ *c*^1/(1+*zν*)^. The slower the quenching process, the closer the crossover point *ε*(*t*_*c*_) comes to the critical point.

The adiabatic-impulse approximation drastically simplifies the picture of the nonequilibrium dynamics of the system. In the early stage from *t* = −∞ to −*t*_*c*_ and the later stage from *t*_*c*_ to ∞ of the quenching process, the dynamics is adiabatic (or quasi-static), and the work is equal to the free-energy change. It means that there is no entropy production in these periods. In contrast, the system is completely frozen during the interval −*t*_*c*_ ≤ *t* ≤ *t*_*c*_. The dynamics during this interval is equivalent to the case of the sudden quenching discussed above. Therefore, 〈*e*^−*σ*^〉_*δ*_ for the whole quenching process is determined solely by the dynamics during the impulse interval from −*t*_*c*_ to *t*_*c*_. As a consequence, the same analysis for sudden quenching processes given above can be applied for the finite-speed quenching processes, but with *ε*_0_ replaced by *ε*(*t*_*c*_) ~ *c*^1/(1+*zν*)^.

## Conclusion

We have found that, near a critical point, for a given tolerance parameter *δ* the ensemble average of *e*^−*σ*^ follows a scaling law in terms of a universal combination of the reduced coupling constant *ε*_0_ of the initial state and the system size *L*: 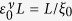 with *ν* being the critical exponent of the correlation length *ξ*_0_. As noted previously^18^,^19^,^20^,^21^,^22^,^23^,^24^,^25^,^26^,^27^,^28^, the Jarzynski equality may break down for many practical and intrinsic reasons. Its breakdown in our case is peculiar as the deviation is determined by an universal combination of *L* and *ε*_0_, which is inherited from the equilibrium scaling behavior of second-order phase transitions. It is stressed that such a universal scaling behavior is not limited to the sudden quenching but holds in general due to the critical slowing down. Our findings may provide a unique application of the Jarzynski equality to study the dynamical properties of phase transitions.

## Additional Information

**How to cite this article**: Hoang, D.-T. *et al.* Scaling Law for Irreversible Entropy Production in Critical Systems. *Sci. Rep.* **6**, 27603; doi: 10.1038/srep27603 (2016).

## Supplementary Material

Supplementary Information
