# Supplementary material for: Scaling Law for Irreversible Entropy Production in Critical Systems
Source: Sci Rep. 2016 Jun 9;6:27603. doi: 10.1038/srep27603 (PMC4899778; doi:10.1038/srep27603)
Supplement: Supplementary Information [file srep27603-s1.pdf]

Scaling Law for Irreversible Entropy Production in Critical Systems: Supplementary Information

Danh-Tai Hoang,[1,2,3] B. Prasanna Venkatesh,[1,4,5] Seungju Han,[6]
Junghyo Jo,[1,7] Gentaro Watanabe,[1,8,9,10] and Mahn-Soo Choi[6,*]

[1] *Asia Pacific Center for Theoretical Physics (APCTP), Pohang, Gyeongbuk 37673, Korea*
[2] *National Institute of Diabetes and Digestive and Kidney Diseases, National Institutes of Health, Bethesda, Maryland 20892, USA*
[3] *Department of Natural Sciences, Quang Binh University, Dong Hoi, Quang Binh 510000, Vietnam*
[4] *Institute for Quantum Optics and Quantum Information of the Austrian Academy of Sciences, Technikerstraße 21a, Innsbruck 6020, Austria*
[5] *Institute for Theoretical Physics, University of Innsbruck, A-6020 Innsbruck, Austria*
[6] *Department of Physics, Korea University, Seoul 02841, Korea*
[7] *Department of Physics, Pohang University of Science and Technology (POSTECH), Pohang, Gyeongbuk 37673, Korea*
[8] *Center for Theoretical Physics of Complex Systems, Institute for Basic Science (IBS), Daejeon 34051, Korea*
[9] *University of Science and Technology (UST), 217 Gajeong-ro, Yuseong-gu, Daejeon 34113, Korea*
[10] *Department of Physics, Zhejiang University, Hangzhou, Zhejiang 310027, China*

(Dated: May 13, 2016)

In the main text, we introduced two different tolerance schemes: One defined by

$$\mathcal{H}(\rho,\delta) = \{\mathbf{S} \mid \rho(\mathbf{S}) > \rho_{\text{cut}}(\rho,\delta)\}, \tag{S1a}$$

$$\sum_{\mathbf{S}\in\mathcal{H}(\rho,\delta)} \rho(\mathbf{S}) = 1-\delta \tag{S1b}$$

in terms of the *microscopic* spin configurations $\mathbf{S}$ and the other defined by

$$\mathcal{I}(P,\delta) = \{S \mid P(S) > P_{\text{cut}}(P,\delta)\}, \tag{S2a}$$

$$\int_{\mathcal{I}(P,\delta')} dS\, P(S) = 1-\delta \tag{S2b}$$

in terms of the *macroscopic* magnetization S (per spin). Here we establish a relation between the two schemes based on the Ginzburg-Landau (GL) theory, which is a natural way to describe the system close to the critical point.

In order to make the point clearer, we further adopt the Gaussian approximation [1]

$$Z \approx \int \prod_{j=1}^{N} d\psi(\mathbf{x}_j)\, e^{-\beta H} \tag{S3}$$

with the GL functional

$$\beta H = \frac{1}{2}\sum_{ij}[\psi(x_i)-M]\chi^{-1}(\mathbf{x}_i-\mathbf{x}_j)[\psi(\mathbf{x}_j)-M], \tag{S4}$$

where $M \equiv \langle\psi(\mathbf{x}_j)\rangle$ is the equilibrium magnetization *per site*. Here N is the number of *coarse-grained* lattice sites rather than the original lattice. However, both being macroscopic, we do not distinguish them here. We define the Fourier transforms of the field and the susceptibility by

$$\psi(\mathbf{x}_j) = \sum_{\mathbf{k}} e^{i\mathbf{k}\cdot\mathbf{x}_j}\psi_{\mathbf{k}} \tag{S5}$$

and

$$\chi(\mathbf{x}) = \frac{1}{N}\sum_{\mathbf{k}} e^{i\mathbf{k}\cdot\mathbf{x}}\chi(\mathbf{k}), \tag{S6}$$

respectively. Recall that

$$\psi_{\mathbf{k}}^* = \psi_{-\mathbf{k}} \in \mathbb{C} \tag{S7}$$

as $\psi(\mathbf{x}_j)\in\mathbb{R}$. The GL functional reads as

$$\beta H = \frac{1}{2}N\frac{(\psi_0-M)^2}{\chi(0)} + N{\sum_{\mathbf{k}}}'\frac{\psi_{\mathbf{k}}^*\psi_{\mathbf{k}}}{\chi(\mathbf{k})}, \tag{S8}$$

where the primed sum means that

$${\sum_{\mathbf{k}}}' \equiv \sum_{\{\mathbf{k}|k_x>0\}}. \tag{S9}$$

We introduce real fields $\varphi_{\mathbf{k}}\in\mathbb{R}$ and rewrite the complex fields ψ_k in terms of them as

$$\psi_0 = \varphi_0\,,\quad \psi_{\mathbf{k}} = \frac{\varphi_{\mathbf{k}}+i\varphi_{-\mathbf{k}}}{\sqrt{2}}\quad \{\mathbf{k}|k_x>0\} \tag{S10}$$

Then the GL functional has the form

$$\beta H = \frac{N}{2}\sum_{\mathbf{k}}\frac{(\varphi_{\mathbf{k}}^2-M\delta_{\mathbf{k},0})^2}{\chi(\mathbf{k})}. \tag{S11}$$

Finally, by rescaling each order parameter component $\varphi_{\mathbf{k}}$ by $\chi(\mathbf{k})$, the distribution function $\rho(\boldsymbol{\phi})$ takes the typical isotropic Gaussian form

$$\rho(\boldsymbol{\phi}) = \frac{e^{-N(\boldsymbol{\phi}-\boldsymbol{\mu})^2/2}}{Z} \tag{S12}$$

with

$$Z = \int\frac{d^N\boldsymbol{\phi}}{(2\pi/N)^{N/2}}\,e^{-N(\boldsymbol{\phi}-\boldsymbol{\mu})^2/2} \tag{S13}$$

* choims@korea.ac.kr

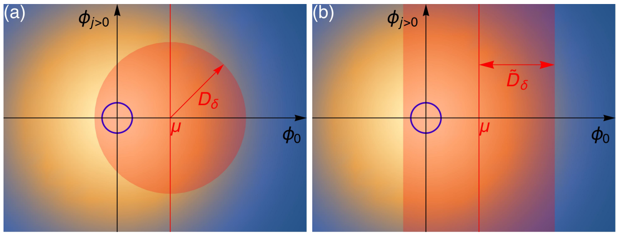

FIG. S1. The tolerance schemes in terms of (a) the distribution function $\rho(\boldsymbol{\phi})$ of microscopic spin configurations $\boldsymbol{\phi}$ and (b) the distribution function $P(\phi_0)$ of rescaled magnetization $\phi_0 = S/\chi(0)$. The background density plot represents $\rho(\mathbf{S})$, the horizontal axis ϕ_0, the vertical axis any other component ϕ_j ($j > 0$), and the small blue circle the N-dimensional hypersphere corresponding to $\rho(\mathbf{S})/\rho(0) = 1/\sqrt{e}$. The red shaded disk and stripe in (a) and (b), respectively, schematically denote the tolerance surviving regions in corresponding tolerance schemes.

where

$$\boldsymbol{\phi} \equiv \begin{bmatrix} \varphi_0/\chi(0) \\ \varphi_{\mathbf{k}_1}/\chi(\mathbf{k}_1) \\ \vdots \\ \varphi_{\mathbf{k}_{N-1}}/\chi(\mathbf{k}_{N-1}) \end{bmatrix}, \quad \boldsymbol{\mu} \equiv \begin{bmatrix} M/\chi(0) \\ 0 \\ \vdots \\ 0 \end{bmatrix} \tag{S14}$$

The tolerance scheme in Eq. (S1) [see Fig. S1 (a)] defines an N-dimensional hypersphere with radius D_δ given by

$$\delta = Q_{N/2}(ND_\delta^2/2) \equiv 1 - \int_{|\boldsymbol{\phi}|<D_\delta} \frac{d^N\boldsymbol{\phi}}{(2\pi/N)^{N/2}}\, e^{-N\boldsymbol{\phi}^2/2} \tag{S15}$$

where $Q_z(\zeta)$ is the *regularized incomplete Gamma function*

$$Q_z(\zeta) \equiv \frac{1}{\Gamma(z)} \int_\zeta^\infty dt\, t^{z-1} e^{-t}\,. \tag{S16}$$

Note that $D_\delta \simeq 1$ for $N \gg 1$ unless $\delta \to 0$ or $\delta \to 1$. On the other hand, the tolerance scheme in Eq. (S2) [see Fig. S1 (b)] defines an N-dimensional slab with thickness $2\tilde{D}_\delta$ given by

$$\delta = 1 - \mathrm{erf}(\tilde{D}_\delta/\sqrt{2}) \tag{S17}$$

because

$$P(\phi_0) \equiv \int \prod_{j\neq 0} \rho(\boldsymbol{\phi}) = \frac{e^{-N(\phi_0-\mu_0)^2/2}}{\sqrt{2\pi/N}}. \tag{S18}$$

In addition, the relation between the two tolerance schemes discussed above also justifies the Monte Carlo simulation, which inevitably samples only finite number of microscopic spin configurations. Suppose that the Monte Carlo sampling has error δ_{MC}. Then the distribution function $\rho_{\text{MC}}(\boldsymbol{\phi})$ simulated by the Monte Carlo method is defined on the space

$$\{\boldsymbol{\phi} \mid |\boldsymbol{\phi} - \boldsymbol{\mu}| < D_{\delta_{\text{MC}}}\} \tag{S19}$$

The distribution function $P_{\text{MC}}(S)$ is extracted from $\rho_{\text{MC}}(\boldsymbol{\phi})$ by

$$\begin{aligned} P_{\text{MC}}(\phi_0) &= \frac{1}{1-\delta_{\text{MC}}} \int_{|\boldsymbol{\phi}-\boldsymbol{\mu}|<D_{\delta_{\text{MC}}}} d\phi_1 \cdots d\phi_{N-1}\, \rho(\boldsymbol{\phi}) \\ &= \frac{e^{-N(\phi_0-\mu_0)^2/2}}{\sqrt{2\pi/N}} \frac{1 - Q_{(N-1)/2}\left((D_{\delta_{\text{MC}}}^2 - \phi_0^2)N/2\right)}{1-\delta_{\text{MC}}} \end{aligned} \tag{S20}$$

Assuming δ_{MC} reasonably small, for large N, the correction factor is a flat function of ϕ_0 and $P_{\text{MC}}(\phi_0)$ is very close to the true $P(\phi_0)$. Therefore, as long as we use the macroscopic tolerance scheme in Eq. (S2), the Monte Carlo simulation provides sufficient sampling.

[1] D. J. Amit, J. Phys. C **7**, 3369 (1974).
